# Reduction of Sleep Medications via a Combined Digital Insomnia and Pharmacist-Led Deprescribing Intervention: Protocol for a Feasibility Trial

**DOI:** 10.2196/47636

**Published:** 2023-07-20

**Authors:** Adam D Bramoweth, Caroline E Hough, Amanda D McQuillan, Brittany L Spitznogle, Carolyn T Thorpe, James J Lickel, Monique Boudreaux-Kelly, Megan E Hamm, Anne Germain

**Affiliations:** 1 Mental Illness Research, Education and Clinical Center VA Pittsburgh Healthcare System Pittsburgh, PA United States; 2 Center for Health Equity Research and Promotion VA Pittsburgh Healthcare System Pittsburgh, PA United States; 3 Pharmacy Services VA Pittsburgh Healthcare System Pittsburgh, PA United States; 4 Eshelman School of Pharmacy University of North Carolina Chapel Hill, NC United States; 5 Behavioral Health William S Middleton Memorial Veterans' Hospital Madison, WI United States; 6 StatCore VA Pittsburgh Healthcare System Pittsburgh, PA United States; 7 Division of General Internal Medicine University of Pittsburgh School of Medicine Pittsburgh, PA United States; 8 Noctem Health Inc Pittsburgh, PA United States

**Keywords:** insomnia, sedatives and hypnotics, mHealth, deprescribing, cognitive behavioral therapy, clinical pharmacist, veterans

## Abstract

**Background:**

Chronic insomnia is one of the most common health problems among veterans and negatively impacts their health, function, and quality of life. Although cognitive behavioral therapy for insomnia (CBT-I) is the first-line recommended treatment, sedative-hypnotic medications remain the most common. Sedative-hypnotics, however, have mixed effectiveness, are frequently prescribed longer than recommended, and are associated with numerous risks and adverse effects that negatively impact veteran function. Meeting the treatment needs of veterans impacted by insomnia requires delivering gold standard behavioral care, like CBT-I, and the reduction of sedative-hypnotics through innovative methods.

**Objective:**

The objective of this feasibility clinical trial is to test a digital CBT-I approach combined with deprescribing to improve the success of sedative-hypnotic reduction among veterans. The intervention combines Noctem Health Clinician Operated Assistive Sleep Technology (COAST), an effective and efficient, scalable, and adaptable digital platform to deliver CBT-I, with clinical pharmacy practitioner (CPP)–led deprescribing of sedative-hypnotic medications.

**Methods:**

In this nonrandomized single-group clinical trial, 50 veterans will be recruited and enrolled to receive CBT-I delivered via Noctem COAST and CPP-led deprescribing for up to 12 weeks. Assessments will occur at baseline, posttreatment, and 3-month follow-up. The aims are to (1) assess the feasibility of recruiting veterans with chronic sedative-hypnotic use to participate in the combined intervention, (2) evaluate veterans’ acceptability and usability of the COAST platform, and (3) measure changes in veterans’ sleep, sedative-hypnotic use, and function at baseline, posttreatment, and 3-month follow-up.

**Results:**

The institutional review board approved the study in October 2021 and the trial was initiated in May 2022. Recruitment and data collection began in September 2022 and is anticipated to be completed in April 2024. Aim 1 will be measured by tracking the response to a mail-centric recruitment approach using electronic medical records to identify potentially eligible veterans based on sedative-hypnotic use. Aim 2 will be measured using the Post-Study System Usability Questionnaire, assessing overall usability as well as system usefulness, information quality, and interface quality. Aim 3 will use the Insomnia Severity Index and sleep diaries to measure change in insomnia outcomes, the Patient-Reported Outcome Measurement Information System Profile to measure change in physical function, anxiety, depression, fatigue, sleep disturbance, participation in social roles, pain, cognitive function, and self-reported sedative-hypnotic use to measure change in dose and frequency of use.

**Conclusions:**

Findings will inform the utility of a combined digital CBT-I and CPP-led deprescribing intervention and the development of an adequately powered clinical trial to test the effectiveness in a diverse sample of veterans. Further, findings will help inform potential new approaches to deliver care and improve access to care for veterans with insomnia, many of whom use sedative-hypnotics that may be ineffective and increase the risk for negative outcomes.

**Trial Registration:**

ClinicalTrials.gov NCT05027438; https://classic.clinicaltrials.gov/ct2/show/NCT05027438

**International Registered Report Identifier (IRRID):**

DERR1-10.2196/47636

## Introduction

### Background

Chronic insomnia is one of the most common health problems among veterans and has a significant negative impact on health, function, and quality of life [1-3]. Insomnia impacts over 50% of veterans [2,4] and contributes to functional impairment, fatigue, reduced alertness, and impaired memory, attention, concentration, and work performance [5-8]. Insomnia also negatively affects comorbid health problems and increases the risk of depression, anxiety, substance use, chronic pain, cardiometabolic disorders, and suicidal behaviors [9-11]. In the general population, COVID-19 pandemic continues to cause significant stress and worry about health and has been associated with increased rates of insomnia as high as 38% and concurrent increases in prescriptions for sedative-hypnotic medications (eg, non–benzodiazepine receptor agonists, such as zolpidem, eszopiclone, zaleplon], temazepam, trazodone) [12-14].

Nonpharmacological treatments of insomnia are the first-line recommended treatment for insomnia. However, sedative-hypnotics (eg, non–benzodiazepine receptor agonists known as z-drugs, such as temazepam and trazodone) are the most common treatment for insomnia, ranging from 5% to 14% in large cohort studies, and even higher rates in survey studies (upward of 25%) [15,16]. Sedative-hypnotic use is associated with increased risks, functional impairment, and negative quality of life and health outcomes [14-16]. Daytime fatigue, impaired cognitive and psychomotor functioning, falls and fractures [17], as well as rare, yet serious, events such as sleep-driving, are linked with the use of sedative-hypnotics [18]. Although considered effective for short-term treatment of insomnia, meaning a few weeks to months, these medications are often taken well beyond 1 year [19,20] despite epidemiological research indicating sleep medications taken for at least one year may be related to elevated incidence of cancer and an increased risk of death (controlling for prior cancer diagnosis) [19]. Use of sedative-hypnotics, especially benzodiazepines, can result in tolerance, dependence, or abuse [20]. Furthermore, these medications only treat insomnia symptoms rather than underlying causal factors, and discontinuation often results in rebound insomnia [20]. Importantly, many patients prefer to avoid sedative-hypnotics if an alternative treatment is available [21]. In sum, sedative-hypnotics are the most common treatment for insomnia by a wide margin, [16,22] but are not the recommended first-line treatment for chronic insomnia [3,23,24].

The state of the science specifies that behavioral interventions, like cognitive behavioral therapy for insomnia (CBT-I), are the first line treatment for chronic insomnia [3,23,24]. Studies within and outside the Veterans Health Administration (VHA) demonstrate that CBT-I significantly improves insomnia symptoms, nighttime sleep quality, and daytime function [25-27]. CBT-I is a multicomponent evidence-based psychotherapy that includes stimulus control [28], sleep restriction [29], cognitive therapy [30], sleep hygiene [31], and relaxation [32]. CBT-I is typically 5-8 sessions (face-to-face; in-person or telehealth) delivered by psychologists or other mental health providers [33]. Randomized controlled trials and meta-analyses have established CBT-I as effective for adults with primary insomnia [34] and those with comorbid insomnia [35], including psychiatric disorders (eg, depression and anxiety [35-37]) and medical disorders (eg, chronic pain [38]). A Veterans Affairs (VA) study with nearly 700 veterans found that 60% who completed CBT-I achieved a treatment response (ie, reduction ≥8 points per the Insomnia Severity Index; ISI). These veterans had a mean ISI change of 20.7 to 10.9, a large effect size (pre- to posttreatment Cohen *d*=2.3) [26]. In terms of long-term treatment gains, a recent review found 50% of patients responded to CBT-I and maintained treatment effects for 4-10 years [39]. CBT-I is also more effective than sedative-hypnotics at reducing sleep onset latency and is equally effective at improving wake after sleep onset and sleep quality [40]. It is important to note that combining CBT-I and sedative-hypnotics may not improve outcomes beyond CBT-I alone [41].

Considering the recommendation of behavioral interventions like CBT-I compared to the high use of sedative-hypnotics and risks involved, it is vital to consider methods to decrease sedative-hypnotic use and increase engagement in CBT-I. Deprescribing is the reduction or withdrawal of a medication managed by a health care professional that aims to reduce harm and improve outcomes [42,43]. Deprescribing interventions to reduce sedative-hypnotic use range from patient-centered approaches (eg, written instructions, relaxation strategies, therapy) to provider education and training [44,45]. A typical intervention will involve gradual dose reduction (GDR), or tapering, and is sometimes accompanied by psychological treatment, like CBT-I [45]. GDR and tapering approaches, including the use of telehealth [46,47], often involve dose reductions of 25%-50% every 1-3 weeks until cessation is achieved [44]. Cessation, however, has varying rates of success (27%-80%). Adding psychological treatments (eg, CBT-I) helps to reduce rebound insomnia and improve cessation rates compared to routine care (odds ratios [OR] 3.38-5.96). However, the results of combining medication tapering and psychological interventions have been mixed [47-49]. Two key challenges to deprescribing sedative-hypnotics are rebound insomnia symptoms and the lack of personalized care (eg, educational brochures) [49]. Many individuals report being unaware of the risks associated with sedative-hypnotics, thus education and multidisciplinary collaboration are critical, especially as these factors are associated with enhanced deprescribing outcomes [50]. Considering these barriers and prior research on CBT-I and sedative-hypnotic tapering [46], a personalized approach that can effectively combine CBT-I and deprescribing has the promise to improve insomnia and sedative-hypnotic outcomes among veterans.

Access to both deprescribing and behavioral interventions would provide maximum impact for veterans; however, access to both interventions remains a barrier. Sedative-hypnotic use is highly prevalent, with many chronic users [16,19,49,51]. While medication prescribers and clinical pharmacists can provide patient education and lead tapering efforts, they do not have training in behavioral insomnia interventions. Despite strong evidence of improved sleep quality and daytime function and with minimal adverse effects and long-term treatment gains [3], CBT-I continues to have limited availability. Even with significant training dissemination efforts by the VA (>1100 trained since 2011), there is still a shortage of trained CBT-I clinicians, especially outside of urban VA medical centers. Additional barriers involve the location of CBT-I delivery, often in mental health clinics, which remains stigmatized for many veterans. Of note, many trained CBT-I clinicians are generalist therapists and insomnia care represents only a small portion of their caseload. CBT-I, like all evidence-based psychotherapies, requires regularly scheduled visits (in-person or telehealth), usually weekly or biweekly, which are not always possible for veterans who work, have caretaking responsibilities, or have transportation challenges.

Digital sleep therapeutics offer personalized, efficient, effective, and scalable treatment for insomnia treatment. Digital CBT-I mobile apps are available, including several developed by the VA, that have similar sleep outcomes as in-person CBT-I [52]. However, these mobile apps are self-management programs—patients do not engage with clinicians via the app—that focus only on improving insomnia symptoms. There are no remote or digital interventions that combine personalized sedative-hypnotic tapering with CBT-I. The Clinician Operated Assistive Sleep Technology (COAST) platform (Noctem Health) offers capabilities for supervised clinical care that allows clinicians to prospectively monitor patients’ symptoms, progress, and adherence to clinician-driven treatment recommendations and to deliver personalized interventions remotely. Thus, COAST can fill the gap for veterans who can benefit from a combined approach of deprescribing and CBT-I with direct and just-in-time supervision, albeit remote, of their care management team.

The COAST platform, unlike other digital insomnia mobile apps, is a decision support tool for clinicians that offers personalized and responsive evidence-based recommendations for each individual patient [53,54]. Data from the initial validation trial and recent quality improvement data from military treatment facilities show that CBT-I delivered via COAST significantly decreases insomnia symptoms per sleep diaries and self-report measures (eg, ISI) to a greater extent than a recent in-person CBT-I trial with active duty military [55]. COAST has been iteratively refined through testing with military personnel, veterans, health care providers, and clinical partners in the Department of Defense Military Health System and academic medical settings [54]. While COAST’s focus is on behavioral sleep assessments and interventions, the platform is flexible and can integrate additional interventions, such as clinical pharmacy practitioner (CPP)–led sedative-hypnotic deprescribing, unlike existing insomnia mobile apps. The COAST platform consists of a provider dashboard and a patient-facing mobile app. It offers a safe, no-contact, or remote, intervention. Assisted by the platform’s technology, providers monitor patient-reported behaviors, while a Health Insurance Portability and Accountability Act (HIPAA)–secure messenger allows just-in-time communication between patients and their treatment team. The integrated secure communication feature is uniquely valuable during the deprescribing process as it promotes rapid feedback for potential adverse effects of GDR or tapering. COAST’s algorithms facilitate the detection of disordered sleep patterns based on patient input, resulting in timely clinical decision-making and personalized sleep interventions based on the core components of CBT-I. COAST’s efficiency helps to significantly reduce clinician time involved in treatment to as low as 10 minutes total per patient versus 30-60 minutes per session for in-person and telehealth modalities (typically 5-8 sessions). COAST also promotes participant engagement [56] and reduces burdensome aspects of face-to-face CBT-I by bringing tools and clinical expertise directly to the patient through their mobile device.

### Specific Aims

Chronic insomnia is one of the most common health problems among veterans and negatively impacts their health, function, and quality of life. Although CBT-I is the first-line recommended treatment, sedative-hypnotic medications remain the most common despite mixed effectiveness and associated risks and adverse effects. To address this gap and reduce sedative-hypnotic use, we are conducting a nonrandomized, single-group pilot clinical trial with 3 aims:

1. To assess the feasibility of recruiting veterans with chronic sedative-hypnotic use to participate in a 12-week combined deprescribing and CBT-I intervention, delivered through the COAST platform
2. To assess veteran acceptability and usability of the COAST platform
3. To assess change in veteran sleep, sedative-hypnotic use, and function pre- to postintervention

The results will inform the design of a larger clinical trial to test the effectiveness of a combined deprescribing and CBT-I intervention delivered via COAST versus usual deprescribing care.

## Methods

### Ethics Approval

This study has been approved by the Institutional Review Board (IRB) of the VA Pittsburgh Health Care System (VAPHS) (1638783). It is registered at clinicaltrials.gov (NCT05027438) with methods reported following Standard Protocol Items: Recommendations for Intervention Trials (SPIRIT) guidelines [57]. All protocol changes and amendments have been reviewed and approved by the VAPHS IRB. Protocol deviations are documented in a deviation log and submitted to the IRB as part of the annual renewal. Any reportable events (eg, serious adverse events) are reported according to the VAPHS IRB and Office of Research and Development guidelines. An audit, independent of the principal investigator, study team, or sponsor, is conducted by the VAPHS Research Compliance Officer at least once every 3 years, or upon study closure if no audit has been completed yet.

All participants undergo the informed consent process prior to enrollment. The informed consent process is administered via video telehealth with the study coordinator. Informed consent documents and HIPAA forms are reviewed during the video consent process and then sent to veterans for completion using DocuSign; veterans are not considered enrolled until signed documents are returned to the study coordinator. veterans also have the option to opt-in for their study data to be included in a research repository, which will allow their deidentified data to be used in other research with appropriate approval of the VAPHS IRB; this is optional and does not impact their ability to participate in the clinical trial. Participants are compensated up to US $175 if they complete all study procedures including the baseline assessment (US $50), posttreatment assessment (US $50), 3-month follow-up assessment (US $50), and the brief interview (US $25).

All participant data are kept private and confidential. Data collection will occur through the COAST platform and stored on Noctem’s Amazon Web Services GovCloud secure cloud-based server behind a firewall with a security policy that includes a dedicated server, regular backup of data, use of a monitored network with active security measures, and well-defined role-based access control. No protected health information (PHI) is stored on the COAST platform. After secure data transfer from Noctem to VA, security protocols in place include study data stored on secure dedicated study drives behind the VA firewall and only study team personnel with access to the data. Participants are assigned a unique identification number to ensure their study-related data are deidentified. While some PHI is collected as part of the informed consent process (ie, email address for DocuSign), no PHI will be included in the data analysis and dissemination of findings.

### Participants and Eligibility

Participants will include 50 veterans recruited from VAPHS and affiliated community based outpatient clinics. See Textbox 1 for inclusion and exclusion criteria.

The medications and doses selected for inclusion were based on clinical practice guidelines and medications indicated for the treatment of insomnia (on or off label) [58]. Medications used to treat insomnia but also used to treat other disorders, such as benzodiazepines (eg, clonazepam) and sedating antidepressants (eg, amitriptyline) were not included. In addition, as this is a feasibility pilot trial, the medications were limited to the most commonly prescribed sleep medications [59].

The primary recruitment method will occur via mail. The study team will mail letters to potentially eligible veterans (see Textbox 1) identified from VA medical records. A prescreen list of potentially eligible veterans will be generated by the study’s data analyst through the VA’s corporate data warehouse. Participants with preliminary eligibility will be stratified by age (<65 or ≥65 years), sex (male or female), and race (White or non-White, including American Indian or Alaska Native, Asian, Black or African American, and Native Hawaiian or other Pacific Islander) with an equal number of letters mailed for each group, until a group’s sample is depleted (see Table 1) and recruitment goals are met (n=50 enrolled). If feasible, depending on COVID-19 restrictions, secondary recruitment methods will be in-person (eg, flyers, clinician-assisted) at VAPHS clinics (eg, primary care, behavioral health, and sleep medicine).

**Inclusion and exclusion criteria.**

**Inclusion criteria**

- VA Pittsburgh Health Care System (VAPHS) veterans aged ≥18 years
- Medical record documentation and self-reports of active sedative-hypnotic use ≥3 days/week for ≥3 months, including zolpidem ≤10 mg immediate release, ≤12.5 sustained release; zaleplon ≤20 mg; eszopiclone ≤3 mg; trazodone ≤400 mg; temazepam ≤30 mg; and hydroxyzine ≤100 mg; veterans on higher doses may be eligible on a case-by-case basis
- A desire to reduce or stop using sedative-hypnotics
- Access to a mobile device

**Exclusion criteria**

- A self-report of a medical or psychiatric disorder that would significantly impair participation (eg, cancer, uncontrolled pain, severe depression)
- A self-report of a medical or psychiatric disorder that can be exacerbated by changes in sleep (ie, seizure disorder, psychotic disorders, bipolar I disorder)
- A self-report of an active substance use disorder
- High risk of suicide per the Columbia-Suicide Severity Rating Scale (C-SSRS)
- Currently engaged in an evidence-based, time-limited psychotherapy (eg, prolonged exposure, cognitive processing therapy, cognitive behavioral therapy for depression, and cognitive behavioral therapy for insomnia)

**Table 1:** Estimated initial recruitment sample (N=1435).

	White (n=1287), n			Non-White^a^ (n=148), n	
	Age <65 years	Age ≥65 years	Age <65 years		Age ≥65 years
Female (n=142)	92	23	26		1
Male (n=1293)	405	767	63		58

^a^Non-White included American Indian or Alaska Native, Asian, Black or African American, and Native Hawaiian or other Pacific Islander.

### Interventions

#### Overview

This is a single-group, nonrandomized clinical trial lasting up to 12 weeks with assessments at baseline (T0), posttreatment (T1), and 3-month follow-up (T2). The intervention will combine CPP-led sedative-hypnotic deprescribing with CBT-I, delivered through the COAST platform and managed by a psychologist trained in CBT-I [53].

#### COAST Delivered CBT-I

**Figure 1:**
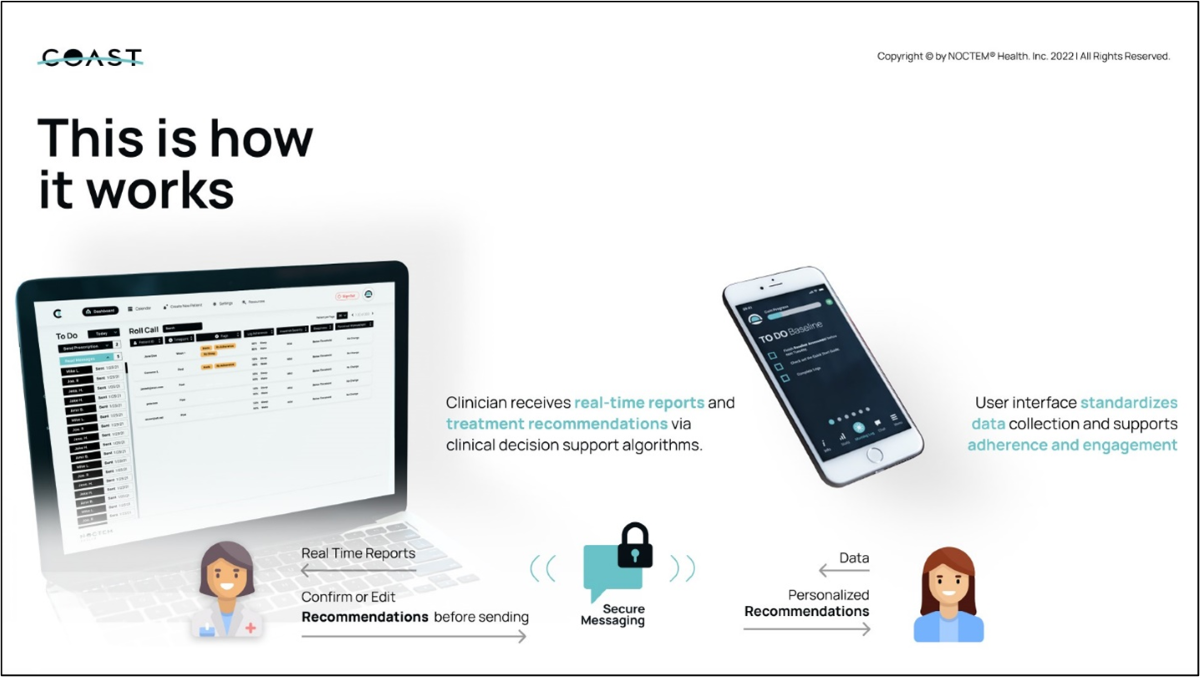
COAST features. COAST: Clinician Operated Assistive Sleep Technology.

The COAST algorithms, based on participant data (eg, sleep diary, self-report measures), will generate treatment recommendations after approximately 1 week of participant data entry [54]. COAST’s treatment recommendations align directly with the core components of CBT-I—stimulus control, sleep restriction, cognitive therapy, and relaxation—with additional strategies targeting nightmares and daytime fatigue as needed. Weekly assessments and daily sleep diaries drive recommendations offered to the clinician. Because COAST is clinical decision support software, the study clinician (ie, a psychologist trained in CBT-I) must review and approve or change algorithm-generated recommendations before they are sent to the participant via the patient-facing mobile app (see Figure 1). This tailored approach allows for modification of COAST’s recommendations based on clinician expertise and knowledge of the participant (eg, a recent stressor that indicates slowing or pausing treatment). To further enhance the treatment process, the clinician is available by secure message (ie, in-platform texting) during business hours to provide rapid support or schedule an appointment as needed (eg, phone, VA Video Connect). The messaging feature also allows for the study team to rapidly notify the participant if daily diaries are incomplete or assessments are missed. As needed, the study team will contact participants by phone or video telehealth for treatment adherence issues. These features offer personalization for each participant, consistent with a case-conceptualization approach based on the veteran’s sleep needs. CBT-I via COAST is typically delivered over 4-6 weeks but has been adapted for this study to last up to 12 weeks as deprescribing can vary and ongoing COAST recommendations may further support deprescribing efforts.

The COAST recommendations are based on CBT-I and the rationale that modifying behaviors impacts the homeostatic and circadian drives. Modifying sleep and daytime behaviors helps regulate wakefulness, which can increase the homeostatic sleep drive and restructures and optimizes sleep and wake times to reinforce the circadian drive. Stimulus control limits the bed for sleep, sexual activity, and sickness, which strengthens the positive association between bed and sleep. Staying in bed while awake and engaging in nonsleep activities perpetuates the cycle of wakefulness, frustration, and arousal. When unable to sleep, it is recommended patients get out of bed and go to another room until sleepiness returns. Sleep restriction, or sleep efficiency training, matches the patients’ sleep opportunity, or prescribed time in bed, to their average total sleep time. This allows the homeostatic and circadian drives to become better aligned. A consistent wake time is (1) the most important cue for setting the biological clock, (2) regulates exposure to morning light, and (3) helps increase the homeostatic sleep drive for subsequent nights. Going to bed only when sleepy, but not before a prescribed bedtime, increases the sleep drive and the likelihood of falling asleep quickly. Intrusive thoughts and worry can result in wakefulness and can contribute to cognitive and physiological arousal. Reduction of intrusive thoughts and worry helps to reduce nighttime stress through the identification and challenging of dysfunctional beliefs. Targeted strategies can help to reduce intrusive thoughts at night and the wake or frustration cycle during awakenings. Relaxation exercises help reduce physical and cognitive arousal that can perpetuate wakefulness. Breathing exercises, guided imagery, and progressive muscle relaxation help to reduce arousal and increase readiness to sleep.

#### CPP-Led Deprescribing

Approximately 1 week after CBT-I is initiated in COAST, participants will meet with a CPP to begin deprescribing. For the deprescribing intervention, the study CPPs will develop a personalized taper for each participant based on their medication, dosage, and factors such as anxiety about the taper, duration of use, comorbid disorders, and insomnia severity. The deprescribing plan will be communicated to each participant either through COAST’s secure messaging or verbally (eg, phone, VA Video Connect). The study CPP will, as needed for the personalized taper, place appropriate orders for different doses or new medications in the VA’s medical record system, the Computerized Patient Record System, concurrent with the scope of practice or for the participant’s VAPHS prescribing provider to review or approve of a controlled substance. As needed, the taper can be extended to 12 weeks, and if necessary, participants can be referred to their VAPHS prescribing provider for further deprescribing assistance after the intervention and posttreatment assessment are complete (T1). At any point during the intervention, the taper can be paused if the participant is struggling to adjust or experiences withdrawal symptoms. All withdrawal symptoms will be self-reported through the COAST platform using the secure messaging or the daily sleep diary and will be responded to within 1 business day by a study team member. Outside of business hours (Monday-Friday, 8 AM-4:30 PM), if necessary, participants can contact emergency services.

### Outcomes Measures

#### Demographics and VA Medical Record

On downloading the COAST app, participants will complete a demographic questionnaire recording their biological sex, gender identity, age, time served in military, and combat status. Psychiatric disorders and psychotropic medications will be collected from participants’ VA medical records, as will their service connection—disabilities and disorders caused by military service (0%-100% [60]).

#### ISI Scores

The ISI will serve as the primary sleep outcome. The ISI is a brief measure of nighttime sleep disruption (eg, severity of sleep onset) and daytime impact (eg, interference with function) [61]. The ISI has 7 items scored 0-4, with higher scores indicating greater severity or impairment. The total score (0-28) is categorized as follows: no insomnia (0-7), subthreshold insomnia (8-14), moderate insomnia (15-21), and severe insomnia (22-28). A reduction pre- to posttreatment ≥8 points indicates a treatment response and a posttreatment score ≤7 indicates remission. The ISI demonstrates good psychometric properties, with validity endorsed by its correlation with sleep diaries [62]. The ISI will be assessed at T0, T1, and T2 as well as biweekly during the intervention.

#### Self-Report of Sedative-Hypnotic Medication

Sedative-hypnotics will be measured by participant self-report, as part of a daily entry in COAST: “I took the following sleep medications: Zolpidem (Ambien), Eszopiclone (Lunesta), Zaleplon (Sonata), Trazodone (Desyrel), Temazepam (Restoril), or None.” As medications will be reduced over the course of treatment, veterans will enter the dose of medication as free text. The daily COAST entry will also involve assessments for adverse effects and withdrawal symptoms (ie, the Clinical Institute Withdrawal Assessment–Benzodiazepines; CIWA-B [63]). Outcomes will include posttreatment (T1) and 3-month follow-up (T2) medication dosage relative to starting dosage at baseline (T0), and binary outcomes of a ≥50% reduction (yes or no) and total cessation (yes or no). Furthermore, medication information from participants’ VA health records will be used to help validate their self-report of sedative-hypnotic use throughout the study.

#### Sleep Diary

Additional sleep outcomes will be measured with a daily sleep diary [64]. Sleep diaries provide information about participants’ sleep behaviors, such as bedtime, rise time, total time in bed, total sleep time, sleep onset latency, number of nighttime awakenings, duration of wake after sleep onset, and sleep efficiency. The sleep diary exhibits good psychometric properties and significant correlations with objective data [65]. Sleep diaries will be completed daily from baseline (T0) through posttreatment (T1) and for 7-days at 3-month follow-up (T2). Daily sleep diary data are used by COAST’s algorithms to generate personalized recommendations for each participant.

#### Patient-Reported Outcome Measurement Information System Adult Profile

Secondary clinical and functional measures will come from the Patient-Reported Outcome Measurement Information System Adult Profile (PROMIS) 29+2. This includes measures of physical function, social roles, anxiety, depression, fatigue, sleep disturbance, pain interference, pain intensity, and cognitive function. Each construct is scored individually (4 items scored 1-5 with a total score range of 4-20) except cognitive function (2 items scored 1-5 with a total score range of 2-10), with higher scores indicating more of the construct being measured. All constructs have high internal validity, with a ranging α=.86-.96. Raw scores are translated to a T-score with a mean of 50 and SD of 10. When all constructs are scored together, a preference score is calculated, representing health‐related quality of life ranging from 0 (as bad as dead) to 1 (perfect or ideal health) [66].

#### Post-Study System Usability Questionnaire and Qualitative Interviews

COAST usability and acceptability will be measured with the Post-Study System Usability Questionnaire (PSSUQ) [67], which measures perceived satisfaction with systems such as websites or mobile apps. The PSSUQ has 16 items, scored 1-7 (1=strongly agree, 7=strongly disagree). The overall usability score is the average (total score divided by number of questions answered). The 3 subscales are usefulness (items 1-6), information quality (items 7-12), and interface quality (items 13-16). Lower scores (<3) indicate higher satisfaction and above-average usability. The measure has a high validity score (α=.94). The PSSUQ will be assessed at 2 time points. First, after the initial sleep or wake schedule recommendation is delivered (T0+1 week), as this will give participants a chance to learn the COAST platform and second, at posttreatment (T1), to measure change over time. To further assess usability or acceptability, 15 veterans will be interviewed (via phone or Microsoft Teams; Microsoft Corp) to gain additional context on their COAST user experience and potential changes or new features that could improve COAST. Veterans will be interviewed based on their PSSUQ scores to get a range of perspectives, if available, per score distribution (eg, 5 low, 5 medium, 5 high; see Table 2 for timeline of assessments).

**Table 2:** Timeline of assessments.

Measures	T0^a^	TX^b^	T1^c^	T2^d^
Demographics	✓			
Sleep diary^e^	✓	✓	✓	✓
Sleep medication^f^	✓	✓	✓	✓
Insomnia Severity Index^g^	✓	✓	✓	✓
Patient-Reported Outcome Measurement Information System Profile 29+2	✓		✓	✓
Post Study System Usability Questionnaire^h^	✓		✓	

^a^T0: baseline.

^b^TX: treatment.

^c^T1: posttreatment.

^d^T2: 3-month follow-up.

^e^T0/T1/T2 baseline: twice daily (morning or evening) for 1 week; during treatment: twice daily for up to 12 weeks.

^f^Integrated into daily sleep diary.

^g^Every 2 weeks during treatment.

^h^At baseline and week 1 assessment during treatment.

### Data Collection, Management, and Quality Control

Primary data collection will be through the COAST participant-facing mobile app with additional data collected via the posttreatment qualitative interviews (phone) and VA medical records. All participants will be assigned a unique study identifier that is not related to any personal identifier such as social security or medical record number. During all phases of the study, participants will be encouraged to enter data in a timely fashion with reminders via the messaging feature on COAST as well as phone calls, as needed. Data submitted via COAST are not stored on participants’ personal devices but directly stored on Noctem Health’s NoSQL database, which is located on an Amazon Web Services GovCloud US-East region server. The database and servers adhere to US Government and Department of Defense security standards and use identity and access management (IAM) controls for its infrastructure services, which are limited to appropriate personnel and are continuously reviewed. Data quality control is enhanced by required responses to questions and limited data response ranges to enhance validity. Prior to secure data transfers from Noctem Health to VAPHS, all PHI will be removed from the data set and data will be reviewed for completion, redundancies, and quality. After data transfer, the study’s VA data analysts will review deidentified data to ensure no PHI is included before data are available to the study team for review and analysis. Noctem and VA co-own all the data. The VAPHS study team will have access to the final data set used for analyses. Noctem will retain a copy of the deidentified data set. A limited data set will be created and shared pursuant to a data use agreement appropriately limiting the use of the data set and prohibiting the recipient from identifying or reidentifying (or taking steps to identify or reidentify) any individual whose data are included in the data set.

### Analytic Plan

#### Power Analysis

Given the estimates from prior work on sedative-hypnotic deprescribing and digital insomnia interventions [46,68,69], we can expect an effect size (Cohen *d*) range for pre-post ISI between 1.09 and 2.26 with an average effect size of 1.73 (small, 0.3, medium, 0.5, large, 0.8) [70]. Given 50 enrolled participants and accounting for a 25% dropout (ie, 37 with pre-post data), we will still have 95% power to detect an effect size of 0.65.

#### Aim 1

To determine the feasibility of recruiting veterans to participate in this combined deprescribing and CBT-I intervention, a CONSORT (Consolidated Standards of Reporting Trials) diagram will be used to track the recruitment, enrollment of eligible participants, and their progression through treatment. During recruitment, the number of participants who contact the coordinator, undergo the study screen, and provide consent will be recorded relative to number of letters sent. To track treatment participation, the number of participants who download and activate the COAST app, complete baseline assessments, engage in treatment beyond the initial recommendation, and complete the intervention will be noted. Chi-square analyses will be used to determine group differences (age, sex, and race) in recruitment response and participation rates.

#### Aim 2

Usability and acceptability of COAST will be evaluated by the PSSUQ. Usability or acceptability will be measured early in treatment (T0+1 week) and at posttreatment (T1), as well as change over time (T1–T0+1 week). Change will be assessed using an intention-to-treat (ITT) approach and fitting mixed effects linear models that test change in usability or acceptability (change in PSSUQ scores over the course of treatment) and include a main effect of time (T0+1 week to T1) and random effects for the veteran.

Audio-recorded qualitative interviews will help to gain more nuanced information on COAST usability or acceptability. The interviews will be interpreted using rapid qualitative inquiry [71,72] to ensure that results are delivered in a timely manner to be meaningful to the study. Interviews will be summarized by the study’s qualitative analyst into a template of categories that reflect topics of interest. The summaries will be coded by 2 analysts to develop the basis of content and thematic analyses.

#### Aim 3

Veteran clinical and functional outcomes will be evaluated using an ITT approach. We will fit mixed effects linear models that measure change over the course of treatment for our outcome measures (ISI, sleep diary, PROMIS) that includes a main effect of time (T0-T1, T0-T2, and T1-T2) and include random effects for the veteran (multiple measurements during treatment). Sedative-hypnotic change will be determined by an ITT approach and fitting a mixed methods logistic model that tests for medication dose reduction (≥50%, yes or no) and medication cessation (yes or no) over the course of treatment that includes a main effect of time (T0-T1, T0-T2, and T1-T2) and random effects for the veteran. Additional analyses will compare completers versus noncompleters and responders versus nonresponders.

## Results

This study was approved by the VAPHS IRB in October 2021 with study initiation in May 2022. Recruitment and data collection began in September 2022 and is ongoing. As of June 1, 2023, there were 25 participants enrolled. The study is expected to be completed by summer 2024. Trial results will be posted on clinicaltrials.gov and disseminated through peer-reviewed journals. The study findings will be communicated to the study sponsor, but the sponsor will not be involved in the dissemination of findings.

## Discussion

Sedative-hypnotic use, as a treatment for chronic insomnia, is highly prevalent among veterans and is associated with considerable health and safety risks. Importantly, use of sedative-hypnotics does not align with best practices for treating insomnia or veteran preferences per the VA/Department of Defense Clinical Practice Guidelines [3]. This pilot study offers deprescribing of sleep medications managed by experts (ie, CPPs) while offering personalized, first-line insomnia treatment with CBT-I integrated and managed by a digital sleep platform. The COAST platform is unique, as it enables the efficient and effective delivery of CBT-I with the capability of integrating CPP-led deprescribing to combine these commonly siloed treatments. Patient-centered and personalized care is important to increase the likelihood of successful reduction or cessation of sedative-hypnotics. The combined and personalized care, from the CPP and psychologist, may result in better veteran outcomes compared to separate interventions, that is sedative-hypnotic deprescribing and CBT-I alone. Offering veterans a digital format of care reduces concerns regarding stigma and addresses barriers such as a lack of transportation and allows for the maintenance of social distancing. Notably, in the wake of COVID-19, older veterans (mean age 63.14 years) have demonstrated an increased preference for digital treatment over in-person treatment [73].

Focus on treating insomnia and reducing sedative-hypnotic use is of high value for veterans and their providers. Treatment response and remission of insomnia improve sleep quality, daytime function, and quality of life. Furthermore, treating insomnia reduces symptom severity in comorbid psychiatric disorders (ie, depression, anxiety, and posttraumatic stress disorder), reduces the risk of developing psychiatric disorders (ie, depression and suicidal behaviors), and reduces the risk associated with sedative-hypnotic use.

The long-term objective of this study is to inform the development of an adequately powered clinical trial to test the effectiveness of the combined deprescribing/CBT-I intervention in a diverse sample of veterans. Future clinical trials may also include a comparison of the combined intervention against treatment as usual or the combination of deprescribing and other digital insomnia platforms, such as the VA’s Insomnia Coach. Beyond large-scale clinical trials, additional information is needed regarding cost and cost-effectiveness of using third-party software to support deprescribing and CBT-I delivery, like COAST, within the VA. In addition, implementation and dissemination are vital for success at scale in an integrated health care system like the VA and in the general community. If deemed cost-effective and if scaled appropriately, digital platforms like COAST can help reduce chronic sedative-hypnotic use, improve sleep quality, enhance functional outcomes for patients, and improve the access, efficiency, and effectiveness of delivering evidence-based care for veterans with insomnia.

## Abbreviations

CBT-I: cognitive behavioral therapy for insomnia
CIWA-B: Clinical Institute Withdrawal Assessment–Benzodiazepines
COAST: clinician operated assistive sleep technology
CONSORT: Consolidated Standards of Reporting Trials
CPP: clinical pharmacy practitioner
GDR: gradual dose reduction
HIPAA: Health Insurance Portability and Accountability Act
IAM: identity and access management
IRB: institutional review board
ISI: Insomnia Severity Index
ITT: intention-to-treat
PHI: protected health information
PROMIS 29+2: Patient-Reported Outcome Measurement Information System Profile
PSSUQ: Post-Study System Usability Questionnaire
VA: Veterans Affairs
VAPHS: VA Pittsburgh Health Care System
VHA: Veterans Health Administration

## Data Availability

Consistent with the VA Office of Research and Development Data Management and Access Plan, a limited and deidentified data set generated or analyzed during this study will be available from the corresponding author. Any available data set will be pursuant to a data use agreement appropriately limiting the use of the data and prohibiting the recipient from identifying or reidentifying any individual whose data are included in the data set.
